# Cross-validation of a high-performance liquid chromatography nevirapine plasma assay in a resource-limited setting in Zimbabwe

**DOI:** 10.4102/ajlm.v10i1.1264

**Published:** 2021-07-08

**Authors:** Faithful Makita-Chingombe, Anthony T. Podany, Timothy Mykris, Farai Muzambi, Richard W. Browne, Andrew J. Ocque, Robin DiFrancesco, Lee C. Winchester, Courtney V. Fletcher, Tinashe Mudzviti, Charles C. Maponga, Gene D. Morse

**Affiliations:** 1 International Pharmacology Specialty Laboratory, School of Pharmacy, University of Zimbabwe College of Health Sciences, Harare, Zimbabwe; 2 Antiviral Pharmacology Laboratory, University of Nebraska Medical Center, Omaha, Nebraska, United States; 3 Translational Pharmacology Research Core, Center for Integrated Global Biomedical Sciences, School of Pharmacy and Pharmaceutical Sciences, University at Buffalo, Buffalo, New York, United States

**Keywords:** cross-validation, HIV, root-cause analysis, high-performance liquid chromatography

## Abstract

An international HIV pharmacology specialty laboratory (PSL) was established at the University of Zimbabwe to increase bioanalytical and investigator capacities. Quantitation of plasma nevirapine in samples from the AIDS Clinical Trials Group protocol 5279 was compared between the University of Nebraska Medical Center PSL and the University of Zimbabwe PSL. Both PSLs employed internally developed methods utilising reverse-phase high-performance liquid chromatography with ultraviolet detection. Eighty-seven percent of the cross-validation results exhibited ± 20% difference.

## Introduction

The University of Zimbabwe (UZ) International HIV pharmacology speciality laboratory (IPSL) was established to increase HIV pharmacology-related research and support research investigator capacity in Africa.^1^ The UZ-IPSL performs drug development assay and clinical research specimens analyses, specifically drug-drug interactions, pharmacokinetic and pharmacodynamic data analyses.

The Clinical Pharmacology Quality Assurance programme, which monitors all PSLs within the AIDS Clinical Trials Group, provided technical guidance to and developed a comparative nevirapine validation study for the UZ-IPSL to analyse replicate HIV clinical samples. Validation of the nevirapine bioanalytics method^2^ was essential at the time because of nevirapine use in the sub-Saharan region and results were generated by a reverse-phase high-performance liquid chromatography (HPLC) system using ultraviolet detection with gradient-elution separation.^3,4,5^ This report compares nevirapine bioanalysis at two laboratories: the United States-based, University of Nebraska Medical Center Pharmacology Specialty Laboratory (UNMC-PSL) and the UZ-IPSL. The UNMC-PSL served as the AIDS Clinical Trials Group 5279 (A5279) protocol designated laboratory. The UNMC-PSL, during the time of the A5279 study, participated in the Clinical Pharmacology Quality Assurance proficiency testing (PT) programme to assure nevirapine assay performance and accuracy. Although each PSL validated the nevirapine assay separately, the lack of data comparing the bioanalytical capacity of pharmacology laboratories in low-middle income countries using clinical samples (rather than spiked plasma samples) was the justification for this project.

Therefore, implementation of the project was the last stage in determining UZ-IPSL’s readiness to begin assaying human samples for clinical trials.

## Methods

### Ethical considerations

Data files used in this comparison study were de-identified and Clinical Pharmacology Quality Assurance was blinded to any data associated with samples. This article followed all ethical standards for research without direct contact with human or animal subjects.

### Project design

The UZ-IPSL utilised the A5279 replicate samples (*n* = 95) that were available at the UZ College of Health Sciences Clinical Trial Research Center (UZCHS-CTRC) for cross-validation of nevirapine assays between the UNMC-PSL and the UZ-IPSL. A5279 was a multi-centre, phase III clinical trial investigating the use of short-course rifapentine or isoniazid for the prevention of active tuberculosis in HIV-positive adults.^6^ In A5279, nevirapine pharmacokinetics when co-administered with rifapentine and isoniazid was one of the exploratory objectives. All A5279 primary aliquots collected from the UZ AIDS Clinical Trials Group CTRC were analysed for nevirapine by the protocol designated PSL at the UNMC-PSL.

### A5279 sample collection and processing

Plasma samples for the quantitation of nevirapine concentrations were obtained from A5279 study participants. Blood was collected into potassium ethylenediaminetetraacetic acid collection tubes and transported on ice to the processing laboratory within 1 h where the plasma was separated from cells by centrifugation (1200 × g, 10 min, 4 °C). The plasma aliquots were frozen (–70 °C) and shipped to the UNMC-PSL. The UZCHS-CTRC retained secondary aliquots for the UZ-IPSL cross-validation assay.

### University of Nebraska Medical Center PSL nevirapine assay

A5279 primary plasma aliquots were shipped from UZCHS-CTRC on dry ice to the UNMC-PSL for determination of the nevirapine. The UNMC-PSL nevirapine determination assay had a quantitation range of 25 ng/mL to 10 000 ng/mL. Samples measuring above the quantitation range were diluted and reanalysed using a validated dilution protocol. Solid-phase extraction was utilised to prepare samples for analysis. The UNMC-PSL utilised a Waters e2695 HPLC coupled to a Waters 2489 ultraviolet (Waters, Milford, Massachusetts, United States) detector, which was controlled with Empower 2 (Waters, Milford, Massachusetts, United States) software as the analytical platform. The performance of the assay was compliant with the United States Food and Drug Administration bioanalytical guidelines for method validation.^7^

### University of Zimbabwe International HIV PSL nevirapine assay

Replicate plasma aliquots (*n* = 95) from A5279 available at the UZCHS-CTRC were shipped to the UZ-IPSL for determination of the nevirapine concentration for cross-validation. The UZ-IPSL measured nevirapine using a validated HPLC-ultraviolet assay detailed previously.^2^ The UZ-IPSL chromatographic system consisted of a Shimadzu LC20A HPLC using ultraviolet photodiode array detection (model SPD-M20A) and LabSolutions Software (version 5.8; Kyoto, Japan). The assay quantitation range was 500 ng/mL to 15 000 ng/mL. Samples that were above the quantitation range were diluted and reanalysed. The performance of the assay was compliant with United States Food and Drug Administration bioanalytical guidelines. Peak purity for nevirapine was assessed to confirm the absence of interferences from potential impurities also extracted from the patient’s sample. A5279 samples were assayed over five runs; the two last runs included samples that were above the upper limit of quantification and were diluted (1:8) according to the validated dilution protocol.^2^

### Data management and analysis

The UZ-IPSL nevirapine results were submitted to the AIDS Clinical Trials Group Data Management Center portal using the Data Submission Utility (Frontier Science Foundation Inc., Brookline, Massachusetts, United States). The A5279 study team approved the release of the UNMC A5279 nevirapine concentration results for comparison with the UZ-IPSL results. The original nevirapine concentration data from the UNMC-PSL were used in the specified A5279 protocol pharmacokinetics analysis; no UZ-IPSL derived pharmacokinetics data were used in the per-protocol study analysis. The Data Management Center compiled the data and provided the de-identified data files to the Clinical Pharmacology Quality Assurance PT unit to perform blinded, comparative statistical analyses of the nevirapine results. After examination (Omnibus 2) and finalisation of the data, the analyses of the paired *t*-test, Deming regression and Bland-Altman analyses were completed using Prism GraphPad (version 8.3; San Diego, California, United States) software.

## Results

**FIGURE 1:**
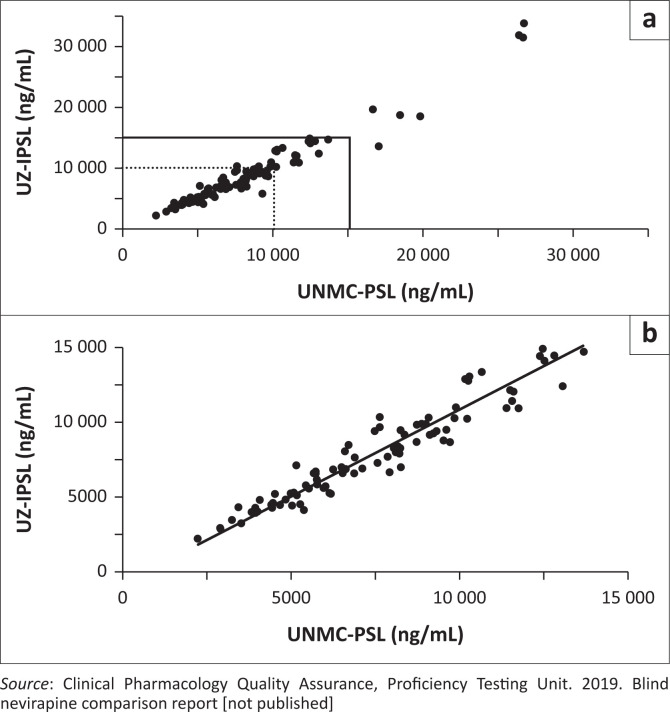
Differences in nevirapine concentrations assayed at UNMC-PSL (Omaha, Nebraska, United States, October 2017) and UZ-IPSL (Harare, Zimbabwe, May 2018); (a) Scatter plot (*n* = 95 data points). (b) Deming regression plot of 87 data points (outliers excluded). Boxes in (a) indicate UNMC upper limit of quantitation (dashed) and UZ-IPLS upper limit of quantitation (solid lines). UZ-IPSL, University of Zimbabwe International HIV Pharmacology Specialty Laboratory; UNMC-PSL, University of Nebraska Medical Center Pharmacology Specialty Laboratory.

Ninety-five replicate plasma aliquots were bioanalysed. The nevirapine concentration range in the analysed samples was ~2000 ng/mL – 34 000 ng/mL (~17-fold). Of this, 97% (*n* = 92) ranged between 2000 ng/mL and 20 000 ng/mL and the remaining 3% (*n* = 3) were above 20 000 ng/mL and not contiguous (Table 1, Figure 1a)^9^. The nevirapine concentration values from the laboratories were tested for normality and only data ≤ 15 000 ng/mL or less were normally distributed. Data 15 000 ng/mL reflected the upper limit of the UZ-IPSL assay and was appropriate because it more closely represented the previously reported nevirapine steady state concentrations (5086 ng/mL – 13 368 ng/mL or 19.1 uM to 50.2 uM).^3,4,5^ Of the data, 93% (*n* = 88) were 15 000 ng/mL or less (Table 1)^9^ while 7% (*n* = 7) were above this value. Therefore, the seven sample pairs were excluded from the method comparison. After exclusion, an additional sample pair was identified as a significant outlier using the Grubbs test and was also excluded.

**TABLE 1:** A comparative study of nevirapine assay results conducted at the University of Zimbabwe International HIV Pharmacology Specialty Laboratory (Harare, Zimbabwe, May 2018) and the University of Nebraska Medical Center Pharmacology Specialty Laboratory (Omaha, Nebraska, United States, October, 2017).

Statistical test	*p*-value	Pearson correlation	Statistical measure of difference	Additional information
**Omnibus 2 for normality**				
(D’Agostino-Pearson)	< 0.0001*	-	K2 = 37.96	Normal nevirapine steady state range: 5086 ng/mL – 13 368 ng/mL
All data points (*n* = 95)	< 0.0001*	-	K2 = 7.072	
Data points ≤ 20 000 ng/mL (*n* = 92)	0.0544	-	K2 = 5.923	UZ-IPSL assay range:500 ng/mL – 15 000 ng/mL
Data points ≤ 15 000 ng/mL (*n* = 88†)	-	-	-	-
Grubbs outlier test	< 0.05*	-	(9309, 5812) 46% difference form average	-
Paired *T*-test	< 0.0001*	-	Mean of differences (UZ-IPSL – UNMC-PSL) = + 430.1	95% confidence = 224.6–635.6
Deming regression	< 0.0001*	-	UZ-IPSL = (1.155* UNMC-PSL) – 718.7	95% confidence slope from 1.077 to 1.234y-intercept from −1231 to −206.8
Bland-Altman, difference	< 0.0001*	*R* = −0.4278 95% confidence = −0.5856–0.2386	UZ-IPSL = (−0.141* UNMC-PSL) + 683 *R*^2^ = 0.183	bias = −43095% confidence: from −2320 to +1460
Bland-Altman, % difference	0.07246	*R* = −0.2692 95% confidence = −0.4541–−0.06205	UZ-IPSL = (−0.001* UNMC-PSL) + 3.48 *R*^2^ = 0.072	bias = −4.488 95% confidence: from −26.79 to +17.81

*Source*: Clinical Pharmacology Quality Assurance, Proficiency Testing Unit. 2019. Blind nevirapine comparison report [not published]

UZ-IPSL, University of Zimbabwe International HIV Pharmacology Specialty Laboratory; UNMC-PSL, University of Nebraska Medical Center Pharmacology Specialty Laboratory.

* , Statistically significant.

† , One data pair in this range was identified as a significant outlier and was excluded from the final analysis.

**FIGURE 2:**
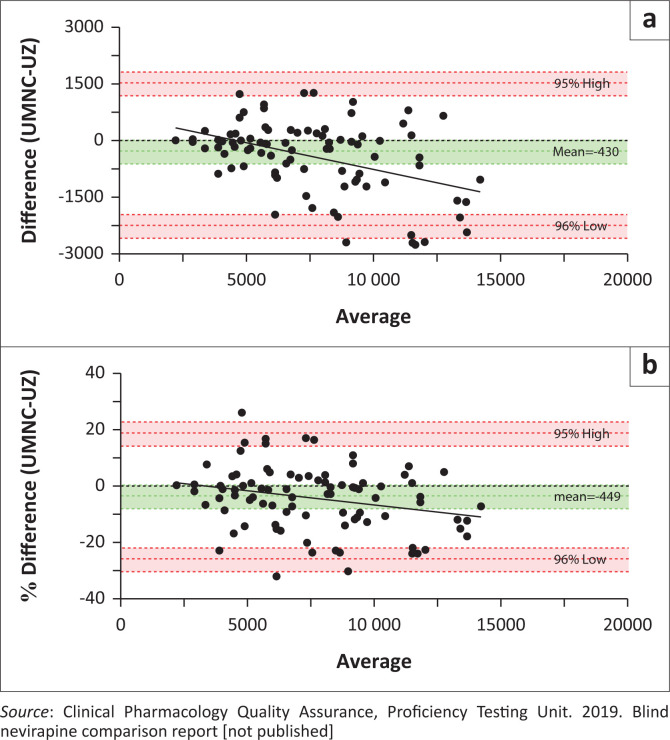
Differences in nevirapine samples (*n* = 87) assayed at UNMC-PSL (Omaha, Nebraska, United States, October 2017) and UZ-IPSL (Harare, Zimbabwe, May 2018). (a) Bland-Altman difference plot indicating proportionally higher difference at high concentrations (mean difference = −430). (b) Bland-Altman percentage difference on normalisation by concentration, correlation not significant (mean % deviation = −4.49). Shaded areas present confidence interval limits for mean (green shade) and agreement limits (red shade). UZ-IPSL, University of Zimbabwe International HIV Pharmacology Specialty Laboratory; UNMC-PSL, University of Nebraska Medical Center Pharmacology Specialty Laboratory.

The two-tailed paired *t*-test of the final data set (*n* = 87) (Table 1)^9^ showed UZ-IPSL nevirapine values were significantly higher with a constant error of 3% – 4% more than UNMC-PSL values. The Deming regression analysis (Figure 1b)^9^ and the Bland-Altman plots (Figure 2)^9^ further confirmed the higher values from the UZ-IPSL. As shown in Figure 2a^9^, regression analysis using the difference plot indicated an increasing proportional difference between the clinical pharmacology laboratories as the concentration of nevirapine increased. However, when the data were normalised by concentration, using the % difference analysis (Figure 2b)^9^, the correlation was no longer significant.

## Discussion

Drug concentrations for nevirapine reported in the literature for HIV-positive individuals indicate a steady state maximum of 5740 ng/mL (5000–7440) and minimum of 3730 ng/mL (3200–5080)^8^. Both nevirapine assays from the two laboratories can detect concentrations that might be below the target concentration for virologic suppression or above the upper limit of the therapeutic range. Although the UZ-IPSL nevirapine concentrations were higher than those of UNMC-PSL and this difference was statistically significant, it is unlikely to have clinical significance. The United States Food and Drug Administration does not provide a window of acceptance for cross-validation studies but allows laboratories to determine a priori the acceptance criteria. Using the established acceptance window for Clinical Pharmacology Quality Assurance PT samples, about 20% of the final target value, and expected concentrations of nevirapine, this difference was acceptable.

The outcomes of the cross-validation also presented an opportunity to perform a root-cause analysis. The UZ-IPSL’s trend of PT outcomes before and during the A5279 sample analysis period was explored. While all PT results were satisfactory, agreement of initial PT sample analyses showed unbiased accuracy, while during subsequent analyses which coincided with A5279 sample analysis, the UZ-IPSL values exhibited a biased, high trend (4% – 19%) as compared to the final target values of that PT round. This shift in bias and accuracy was provided within the PT report and the UZ-IPSL was able to complete a root-cause analysis and remediate as needed.

### Limitations

One limitation to consider is that the calibration ranges for the two equipment sets used for the nevirapine analysis were not identical. Future comparisons of this nature may benefit from evaluations where the calibration range is the same for both laboratories. In addition, limited data points above 20 000 ng/mL entailed difficulties in quantitation of proportional error at that level.

### Conclusion

The cross-validation study provided evidence that the UZ-IPSL performance using a nevirapine assay that was validated in their laboratory was acceptable and correlated with the results of an experienced PSL. This study afforded the UZ-IPSL a valuable opportunity to implement operations using its validated nevirapine assay for the analysis of samples obtained from a clinical research protocol and adopt procedures for handling of protocol specimens based on experience. Furthermore, the outcomes of the cross-validation emphasised the value of PT and provided an occasion to perform root-cause analysis.

## References

[1] Mtisi TJ, Maponga C, Monera-Penduka TG, et al. Strategic establishment of an International Pharmacology Specialty Laboratory in a resource-limited setting. Afr J Lab Med. 2018;7(1):a659. 10.4102/ajlm.v7i1.659 PMC5843799 29535916

[2] Makita-Chingombe F, Ocque AJ, DiFrancesco R, et al. Development and validation of a high performance liquid chromatography method to determine nevirapine in plasma in a resource-limited setting. Afr J Lab Med. 2019;8(1):a880. 10.4102/ajlm.v8i1.880 PMC6556864 31205870

[3] Bolaris MA, Keller MA, Robbins BL, Podany AT, Fletcher CV. Nevirapine plasma concentrations in human immunodeficiency virus-exposed neonates receiving high-dose nevirapine prophylaxis as part of 3-drug regimen. J Pediatric Infect Dis Soc. 2017 3 1;6(1):102–104. 10.1093/jpids/piv084 26803329

[4] Pav JW, Rowland LS, Korpalski DJ. HPLC-UV method for the quantitation of nevirapine in biological matrices following solid phase extraction. J Pharm Biomed Anal. 1999;20(1–2):91–98. 10.1016/S0731-7085(98)00312-4 10704012

[5] Fan-Havard P, Liu Z, Chou M, et al. Pharmacokinetics of phase I nevirapine metabolites following a single dose and at steady state. Antimicrob Agents Chemother. 2013 5;57(5):2154–2160. 10.1128/AAC.02294-12 23459477 PMC3632909

[6] Swindells S, Ramchandani R, Gupta A, et al. One month of rifapentine plus isoniazid to prevent HIV-related tuberculosis. N Engl J Med. 2019;380:1001–1011. 10.1056/NEJMoa1806808 30865794 PMC6563914

[7] United States Food and Drug Administration. Guidance for industry: Bioanalytical method validation. Rockville, MD: FDA; 2001.

[8] Nevirapine PK fact sheet [homepage on the Internet]. 2016 [cited 2020 Mar 19]. https://liverpool-hiv-hep.s3.amazonaws.com/prescribing_resources/pdfs/000/000/059/original/HIV_FactSheet_NVP_2016_Mar.pdf?1520612265

[9] Clinical Pharmacology Quality Assurance, Proficiency Testing Unit. 2019. Blind nevirapine comparison report [not published].

